# Microbial Composition in Larval Water Enhances Aedes aegypti Development but Reduces Transmissibility of Zika Virus

**DOI:** 10.1128/msphere.00687-21

**Published:** 2021-12-08

**Authors:** William Louie, Lark L. Coffey

**Affiliations:** a University of California, Davis, School of Veterinary Medicine, Department of Pathology, Microbiology, and Immunology, Davis, California, USA; Stanford University School of Medicine

**Keywords:** *Aedes aegypti*, Zika virus, arbovirus, microbiome, mosquito, susceptibility, transmission, vector competence

## Abstract

Arthropod-borne viruses comprise a significant global disease burden. Surveillance and mitigation of arboviruses like Zika virus (ZIKV) require accurate estimates of transmissibility by vector mosquitoes. Although *Aedes* species mosquitoes are established as competent ZIKV vectors, differences in experimental protocols across studies prevent direct comparisons of relative transmissibility. An understudied factor complicating these comparisons is differential environmental microbiota exposures, where most vector competence studies use mosquitoes reared in laboratory tap water, which does not represent the microbial complexity of environmental water where wild larvae develop. We simulated natural larval development by rearing Californian Aedes aegypti larvae with microbes obtained from cemetery headstone water compared to conventional tap water. A. aegypti larvae reared in environmental cemetery water pupated 3 days faster and at higher rates. Mosquitoes reared in environmental water were less competent vectors of ZIKV than laboratory water-reared A. aegypti, as evidenced by significantly reduced infection and transmission rates. Microbiome comparisons of laboratory water- and environment water-reared mosquitoes and their rearing water showed significantly higher bacterial diversity in environment water. Despite this pattern, corresponding differences in bacterial diversity were not consistently observed between the respective adult mosquitoes. We also observed that the microbial compositions of adult mosquitoes differed more by whether they ingested a bloodmeal than by larval water type. Together, these results highlight the role of transient microbes in the larval environment in modulating A. aegypti vector competence for ZIKV. Laboratory vector competence likely overestimates the true transmissibility of arboviruses like ZIKV when conventional laboratory water is used for rearing.

**IMPORTANCE** We observed that A. aegypti mosquitoes reared in water from cemetery headstones instead of the laboratory tap exhibited a reduced capacity to become infected with and transmit Zika virus. Water from the environment contained more bacterial species than tap water, but these bacteria were not consistently detected in adult mosquitoes. Our results suggest that rearing mosquito larvae in water collected from local environments as opposed to laboratory tap water, as is conventional, could provide a more realistic assessment of ZIKV vector competence since it better recapitulates the natural environment in which larvae develop. Given that laboratory vector competence is used to define the species to target for control, the use of environmental water to rear larvae could better approximate the microbial exposures of wild mosquitoes, lessening the potential for overestimating ZIKV transmission risk. These studies raise the question of whether rearing larvae in natural water sources also reduces vector competence for other mosquito-borne viruses.

## INTRODUCTION

The global expansion of arthropod-borne viruses (arboviruses) poses a significant public health threat. Climate change and rapid urbanization may accelerate the zoonotic spillover or reemergence of arboviruses, increasing outbreaks in humans (1–3). Zika virus (ZIKV) (*Flaviviridae*, *Flavivirus*), which was understudied since its discovery in 1947 in Uganda (4), garnered worldwide attention following outbreaks in 2015 to 2016 (3, 5, 6). The wave of ZIKV epidemics, accompanied by newly recognized teratogenic phenotypes wherein ZIKV causes adverse outcomes in fetuses from infected pregnant mothers, now referred to as congenital Zika syndrome (7, 8), fueled efforts to better understand and mitigate transmission to curtail disease. Although the ZIKV pandemic of 2015 to 2016 has ended, ZIKV may reemerge via increased numbers of immunologically naive people and the geographic expansion of *Aedes* species vectors (9, 10).

Determining the ability of a mosquito to become infected by and transmit a virus (vector competence) is crucial for guiding surveillance and control, including identifying mosquito species to monitor and eliminate and for modeling outbreak risk. Evaluating vector competence in the laboratory entails exposing mosquitoes to an infectious bloodmeal, followed by the detection of viral RNA or infectious virus in mosquito tissues and saliva after an incubation period usually 3 to 14 days. Mosquito-borne arboviruses must escape the mosquito midgut, infect the salivary glands, and be secreted into saliva for transmission. Although the approach for assessing laboratory vector competence is standard, outcomes across studies vary greatly (6) and may be influenced by virus strain and passage history (11), virus dose (12), mosquito species (13), intraspecies mosquito genetics (14, 15), larval nutrition and competition (16, 17), and incubation temperature (18–20). Since 2017, many vector competence studies have been performed using Aedes aegypti and Aedes albopictus from various geographic origins and post-2015 strains of ZIKV (21–28). The absence of uniformity in the variables involved in laboratory vector competence makes direct comparisons across studies difficult. However, such comparisons are needed to assess reproducibility and identify differences in vector competence across geographies.

The mosquito microbiome is an important variable that influences arbovirus vector competence, wherein specific taxa can modify it. A. aegypti mosquitoes infected with the bacterial endosymbiont *Wolbachia* have a reduced ability to transmit ZIKV, dengue virus (DENV), and chikungunya virus (CHIKV), prompting field trials and experimental releases of *Wolbachia*-infected mosquitoes as a means of population replacement (29). Similarly, the bacterium *Chromobacterium* Csp_P reduces transmission of DENV by A. aegypti and Plasmodium falciparum by Anopheles gambiae (30). Members of the bacterial genus *Asaia* may also confer resistance of mosquitoes to arboviruses and *Plasmodium* (31–33). A. aegypti colonized with *Serratia* bacteria are more susceptible to infection by DENV and CHIKV *in vivo* but less susceptible to ZIKV *in vitro* (34–37). However, the functional roles of specific microbial strains in modulating the vector competence of mosquitoes in nature, where gut microbes exist as a community rather than as a monoculture, remain unclear. Examination of microbial strains in gnotobiotic mosquitoes requires repeatability in a microbial community context, including in the aqueous larval form. To address this gap, we analyzed the microbial structures of larval A. aegypti to elucidate the community dynamics of microbes that colonize larvae and adults, and we then assessed how differences in larval rearing environments and microbial composition affect ZIKV vector competence.

We modified the A. aegypti larval rearing environment by introducing microbes at different diversities and abundances. Since microbes in mosquitoes are primarily acquired through the environment (38, 39), rearing A. aegypti in different water sources provides control of microbial input to A. aegypti colonies in the laboratory (40, 41). Previous work showed that the bacterial microbiota of field-caught *Aedes* mosquitoes varies geographically (42) and that rearing field mosquitoes in a laboratory setting results in a convergence of the gut microbiota in just one generation (43). Moreover, larva-acquired microbes play a significant role in larval development, where axenically (raised as a single organism, free of any microbes) reared mosquitoes exhibit inconsistent pupation success and reduced adult size, likely due to a lack of nutritional supplementation by larval gut microbes (38, 44). Additionally, some larval gut microbes are passed transstadially to adults, suggesting symbiosis through multiple mosquito life stages (45, 46). Consequently, microbes acquired by larvae are expected to influence *Aedes* mosquito physiology and immune status (47–49), which, along with direct physical interactions by microbes, is expected to impact ZIKV vector competence (50, 51). We used larval rearing water that we determined contained a relatively low microbial content compared to microbe-rich water collected from outdoor environments in which A. aegypti larvae are naturally found to determine whether differences in water sources influence ZIKV vector competence in a controlled mosquito genetic background. Our data show that reduced microbial exposure in colonized mosquitoes reared in laboratory water (LW) versus environmental water (EW) modulates vector competence and could explain the variability in vector competence between laboratory and field mosquitoes.

## RESULTS

### Bacterial abundance and diversity decline during A. aegypti larval development.

**FIG 1:**
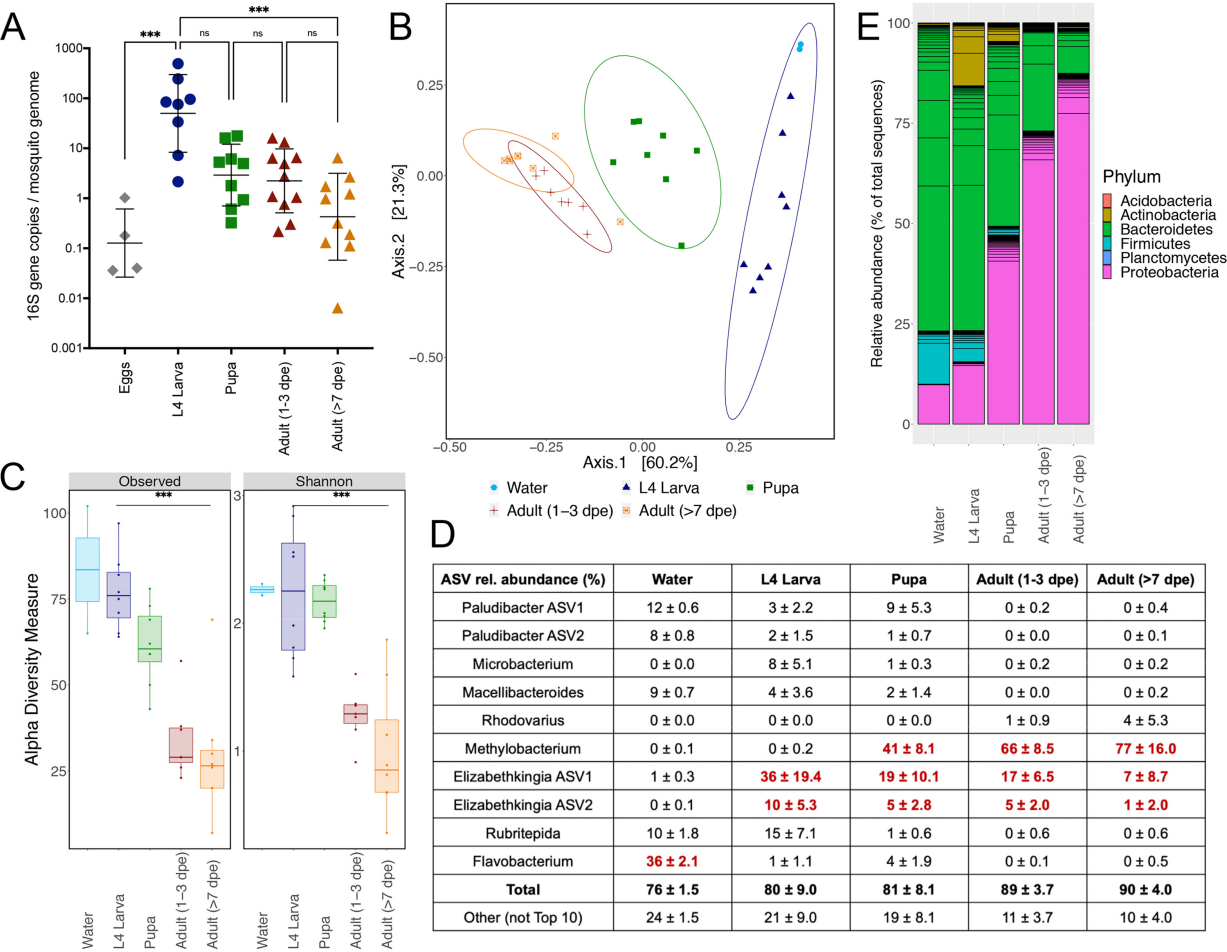
Microbial abundance decreases over the life of a mosquito, although some bacterial taxa persist. (A) Quantification of bacteria at each life stage of A. aegypti from Los Angeles, CA, normalized by the A. aegypti gene RPS17. Each dot represents a single mosquito or a pool of 100 to 200 eggs. dpe, days posteclosion. (B) PCoA at the amplicon sequence variant (ASV) level, by UniFrac distances of microbial composition within each life stage. (C) Alpha diversity of each life stage, using two metrics, observed ASVs and Shannon diversity. (D) Top 10 most abundant ASVs across all samples. Total indicates the sum of the top 10 ASVs, named by genus, while Other indicates the total remaining (non-top 10) ASVs. Red text highlights the ASVs that comprise the most common sequences or ASV types in the sample. (E) Relative abundances of all sequences, by ASV, colored by phylum. Samples for microbiome analysis in panels A to E were as follows: water (*n* = 2, sampled at the larval rearing midpoint [1 week]) and mosquitoes (*n* = 8 per life stage, except 1-3 dpe where *n* = 7; *n* = 4 pools of 100 to 200 eggs). For panels A and C, *** denotes significance at a *P* value of <0.001 using a Kruskal-Wallis test with multiple comparisons. ns, not significant (*P* > 0.05).

We began by assessing bacteria that persist through A. aegypti life stages. Persistence was defined as a bacterial taxon detected in more than one life stage, starting at the larval stage. A total of 31 mosquitoes reared in environmental cemetery water representing 4th-instar larvae (L4) (*n* = 8), pupae (*n* = 8), and adults (1 to 3 days posteclosion [dpe], *n* = 7; >7 dpe, *n* = 8) or pools of 100 to 200 eggs were sampled, and the numbers of bacterial amplicon sequence variants (ASVs) were compared among individuals and to the rearing water (Fig. 1A). Adult mosquitoes were divided into two age classes,1 to 3 dpe and >7 dpe, to compare young and old adults. Bacteria were scarce in washed eggs but significantly increased in L4 larvae (*P* = 0.0008 by a Kruskal-Wallis test). Although the bacterial abundance decreased across the totality of mosquito development (*P* = 0.0002 by a Kruskal-Wallis test), no difference in bacterial abundance between pupae and newly emerged adult females at 1 to 3 dpe (*P* > 0.999 by a Kruskal-Wallis test) was detected, nor was there a difference between young and old adult females at >7 dpe (*P* = 0.7802 by a Kruskal-Wallis test). The bacterial abundance in L4 larvae was significantly lower than that in adult females at 7 dpe, where a decrease in the mean 16S/RPS17 ratio from 129 (geometric mean = 50; geometric standard deviation [SD] = 6) to 1.4 (geometric mean = 0.4; geometric SD = 7) (*P* = 0.0008 by a Kruskal-Wallis test) was detected. A total of 200 ASVs were identified across all life stages (Table 1), with 102 observed in water, 124 in larvae, 125 in pupae, and 99 in adults (see Fig. S1A in the supplemental material). Thirty-one ASVs representing 19 bacterial genera were shared among the rearing water, larvae, pupae, and adults, and most belonged to the phylum *Bacteroidetes* (Fig. S1A and B). The microbial community compositions across life stages were also unique, shown by the distinct clustering of samples by life stage (Fig. 1B). The microbial compositions of larvae clustered close to water, while pupal compositions were more similar to those in adult mosquitoes. Concordant with the decline in microbial abundance and compositional shifts with life stage, a decline in alpha diversity (total observed species and Shannon diversity indices) was also detected, with the greatest difference in alpha diversity between L4 larvae and adults at >7 dpe (*P* = 0.0009 [observed species] and *P* = 0.0036 [Shannon] by a Kruskal-Wallis test) (Fig. 1C). The 10 most abundant ASVs accounted for nearly 80% of the L4 larval bacteria, with the proportion increasing to 90% as adults at >7 dpe (Fig. 1D). While *Flavobacterium* constituted the most common bacterial ASV in the rearing water (36%), *Elizabethkingia* was most common in larvae (two distinct ASVs, totaling 46%), while *Methylobacterium* expanded from 41% during pupation to 77% as adults at >7 dpe. At the phylum level, *Proteobacteria* were progressively significantly enriched with each developmental stage (larva, 16% ± 7%; pupa, 47% ± 9%; adult at 1 to 3 dpe, 73% ± 9%; adult at >7 dpe, 87% ± 13% [*P* < 0.0001 by a Kruskal-Wallis test]), such that they comprised the majority of bacteria in adult mosquitoes despite comprising a smaller relative fraction in the rearing water (10% ± 2%) (Fig. 1E). Taken together, these data show that the microbes in rearing water that colonize A. aegypti are at the highest abundance and diversity at the larval stage and then decrease in relative abundance during development. A fraction of the microbes, primarily in the phyla *Proteobacteria* and *Bacteroidetes*, detected in rearing water persist and are enriched in adult A. aegypti.

**TABLE 1:** 16S data sets used for microbiome analysis^*a*^

Dataset name	ZIKV Bloodmeal	Groups compared	16S region	Total Sample Size	Total # raw reads	Total ASVs (after filtering)	Mean # reads per sample (after filtering)
AM1019LS	none	Life stages(L4, pupa, adult 1-3 dpe/ 7 dpe)	V3-V4	34	19.8 M	200	12,0872
AM1019ZE	PR15	LW vs. EW1*	V3-V4	60	15.6 M	1077	68,030
AM820ZE	BR15	LW vs. EW4	V4	77	17 M	221	5,228

a All data sets included Los Angeles A. aegypti mosquitoes with bacterial DNA from their respective rearing water samples. *,the data set contains samples for LW, EW1, and EW2. Group EW2 was excluded as after filtering; it failed to meet the threshold coverage level of 1,000 reads.

10.1128/msphere.00687-21.1 FIG S1 Bacterial ASVs shared in various life stages. (A) Shared and distinct ASVs detected in rearing water, larvae, pupae, and adult mosquitoes. The text box lists shared bacteria by genus. Red text indicates genera that were also detected in eggs after surface sterilization. (B) Relative abundances of the 19 genera shared among water, larvae, pupae, and adult mosquitoes grouped by bacteria taxa. Download FIG S1, EPS file, 2.9 MB. Copyright © 2021 Louie and Coffey. 2021 Louie and Coffey. https://creativecommons.org/licenses/by/4.0/ This content is distributed under the terms of the Creative Commons Attribution 4.0 International license.

### A. aegypti larvae reared in laboratory water exhibit delayed pupation relative to larvae reared in environmental water.

**FIG 2:**
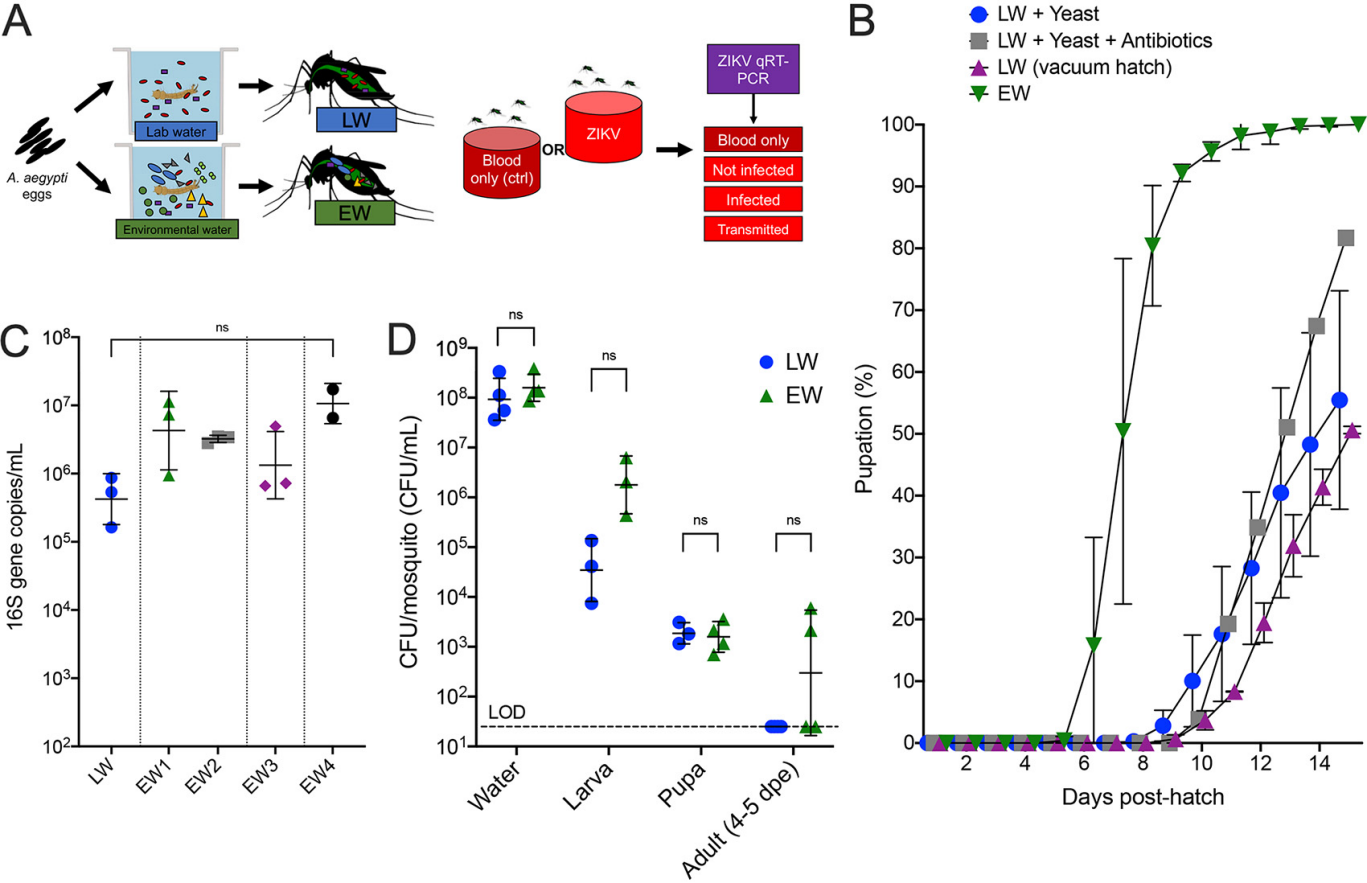
Mosquitoes reared in environmental water develop faster than those reared in laboratory water. (A) Experimental design showing treatment of A. aegypti eggs with either laboratory water (LW) or environmental water (EW) from cemetery headstones. (B) Pupation kinetics and rates relative to the number of larvae that hatched in cohorts of 500 to 600 larvae per liter. LW was spiked with either live baker’s yeast or yeast and antibiotics (penicillin-streptomycin-kanamycin at 50 μg/ml). Each symbol shows the mean cumulative percentage of pupated larvae on that day, with error bars denoting the range. Time course differences in pupation were determined by mixed-effects analysis (one-way ANOVA) with repeated measures and multiple comparisons. Each symbol represents the mean from replicate rearing experiments (*n* = 3). Individual pupation rates for replicate experiments are shown in Fig. S2A in the supplemental material. (C) 16S rRNA qPCR of rearing water at 7 days posthatching. EW was collected on 4 separate occasions (EW1 to -4). Each symbol shows the geometric mean of PCR results from DNA extracted from 200 μl water. Values were compared by a Kruskal-Wallis test with multiple comparisons. (D) Colony counts of bacteria represented as CFU cultured on LB agar at 37°C. Each symbol shows the average from five homogenized mosquitoes or 40 μl of water at the midpoint (7 days posthatch) of a rearing experiment. The absence of colonies detected is reported at the limit of detection (LOD) of 40 CFU/ml. Pairwise comparisons between LW and EW were performed by Mann-Whitney tests.

We next asked whether the source and nature of larval water affected the kinetics and success of larval development. Eggs from colonized A. aegypti were surface sterilized, hatched, and reared to adulthood in standard laboratory water (LW) from the tap or environment water (EW) collected outdoors from cemetery headstones (Fig. 2A). Mosquitoes in both water types were reared at the same density and were supplemented with the same larval food quantity, which was standardized to eliminate differences in food availability and that was also sterilized to avoid introducing additional microbes. Larvae reared in LW exhibited significantly delayed pupation and first pupated on day 8, compared to EW-reared mosquitoes that pupated starting on day 5 (*P* = 0.0005 by a paired *t* test) (Fig. 2B). Furthermore, the percentage (50 to 81%) of larvae that pupated by day 14 in LW was significantly lower than in EW, where 100% of larvae pupated (*P* = 0.0004 by mixed-effect analysis of variance [ANOVA] with multiple comparisons). LW mosquitoes pupated slower than EW mosquitoes, even when the water was supplemented with Saccharomyces cerevisiae (baker’s yeast) with or without antibiotics (adjusted *P* = 0.0023 by mixed-effect ANOVA with multiple comparisons), which is conventionally used to induce hatching via hypoxia (52), and also when vacuum hatching was added, also with the goal of increasing hatch rates (adjusted *P* = 0.0022 by mixed-effect ANOVA with multiple comparisons). This suggests that microorganisms in the environmental water promote pupation success and augment the larval growth kinetics of A. aegypti. We also assessed whether enhanced pupation was associated with a higher bacterial density in EW by comparing the bacterial levels in LW to those in four EW samples (EW1 to -4) collected from the rearing pans at 7 days posthatching. Surprisingly, bacterial DNA quantities in larval pans 7 days after hatching were not significantly different (*P* = 0.078 by a Kruskal-Wallis test) across LW samples or any EW sample (Fig. 2C), suggesting that the total microbial abundance did not influence the differences in the rates of larval development to pupation. Recognizing that gene sequencing does not represent living bacteria, we also cultured bacteria and compared the bacterial densities in LW and EW samples as well as in larvae, pupae, and adults (4 to 5 dpe) reared in both water types. The numbers of bacterial colonies culturable on LB agar were not significantly different between LW and EW (*P* = 0.3143) or between LW- and EW-reared larvae (*P* = 0.1), pupae (*P* > 0.99), or early adults (*P* = 0.4286 [all by a Mann-Whitney test]) (Fig. 2D), further suggesting that the abundance of culturable bacteria does not significantly impact larval development kinetics.

**FIG 3:**
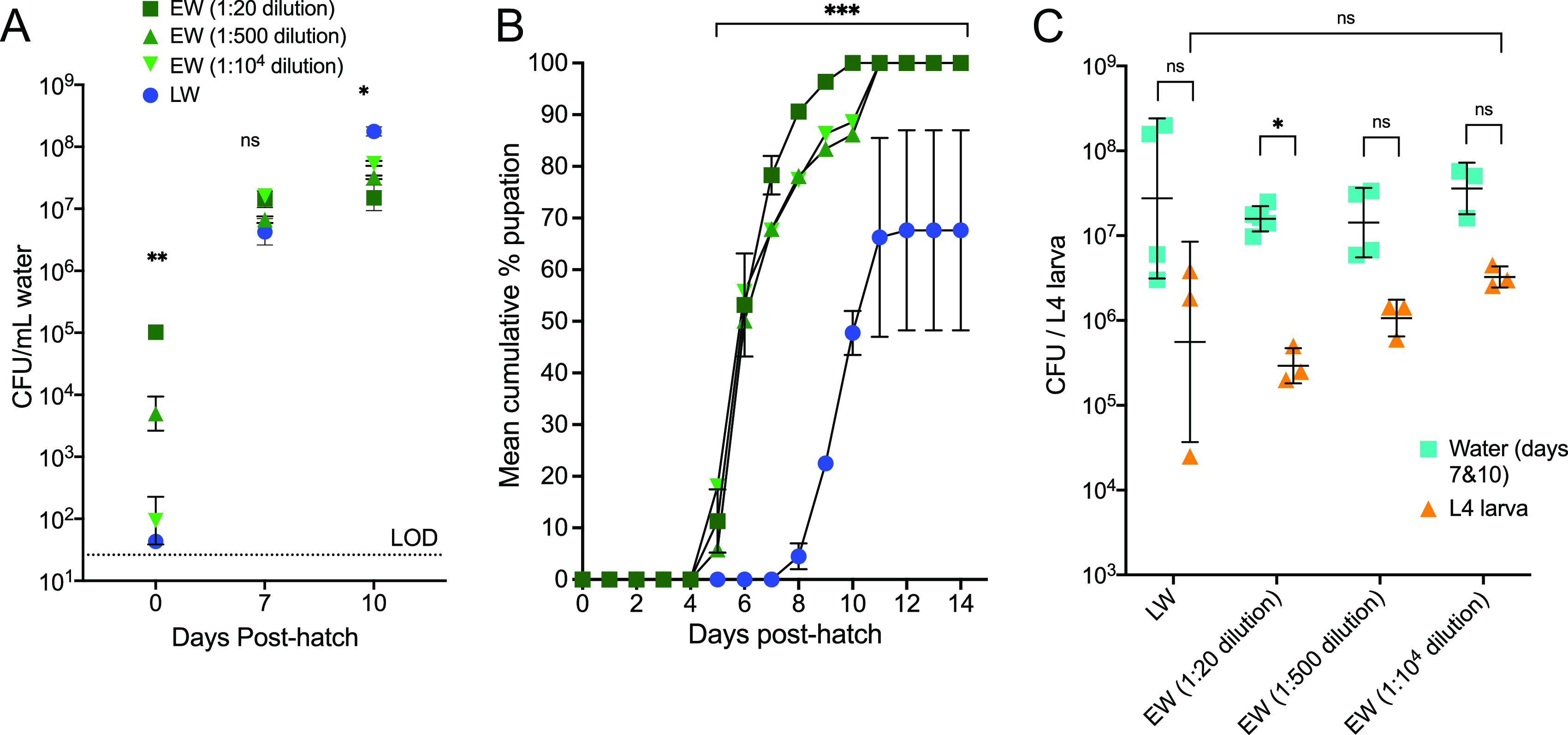
Dilution of microbes pelleted from environmental water does not delay A. aegypti larval development, which is still faster than for larvae reared in laboratory water. Microbes pelleted and washed from 3 liters of EW were stored in glycerol stocks; diluted 1:20, 1:500, and 1:10^4^; and then spiked into LW. (A and B) Bacterial growth for each water treatment (A) and pupation rates (B) were determined. Each symbol in panel B shows the mean cumulative percentage of pupation over time (individual rates for replicate experiments are shown in Fig. S2B in the supplemental material), with error bars denoting the range. Each symbol in panel A shows the geometric mean from triplicate measurements, and error bars denote the geometric standard deviations. Statistical tests were performed using a mixed-effects analysis (one-way ANOVA) with repeated measures and multiple comparisons. (C) Bacterial counts from water at days 7 and 10 were aggregated and are plotted with their respective 4th-instar larvae (L4) that were also sampled at the same time. Pairwise comparisons were performed using the Mann-Whitney test.

10.1128/msphere.00687-21.2 FIG S2 Development times of Los Angeles A. aegypti mosquitoes, plotted by individual experimental replicates. Pupation differences between EW and LW variations in Fig. 2B (A) and LW and EW dilutions in Fig. 3B (B) are shown. Black lines denote the average percentages of pupation from replicate experiments. Download FIG S2, EPS file, 0.2 MB. Copyright © 2021 Louie and Coffey. 2021 Louie and Coffey. https://creativecommons.org/licenses/by/4.0/ This content is distributed under the terms of the Creative Commons Attribution 4.0 International license.

Given that the abundance of bacteria in the larval rearing water did not explain the differences in larval growth and pupation success, we next addressed whether other differences in EW versus LW were influencing mosquito growth. To control for exogenous micronutrient content and water chemistry that could confound the observed differences in larval development, we reared larvae in diluted EW to ablate the potential progrowth effect from EW due to these other factors. EW microbes were pelleted, washed five times in phosphate-buffered saline (PBS), and spiked into LW at different dilutions. Although the colony-forming bacterial quantities of EW dilutions ranged from 10^2^ to 10^5^ CFU/ml at day 0 (comparable at the lowest density to 10^1.5^ CFU/ml in LW), by day 7, the bacterial numbers in all EW dilutions and LW were not significantly different (*P* = 0.1 by a Kruskal-Wallis test) and reached ∼10^7^ CFU/ml (Fig. 3A). The pupation rates were not different (*F* = 5.33 and *P* = 0.07 by mixed-effect ANOVA) regardless of the EW dilution, and all EW groups exhibited 100% pupation by 10 dpe, which was in contrast to pupation from LW, where the mean was 62% (peak of 87%), which was significantly lower than those of all EW dilutions (*F* = 17.19 and *P* = 0.0008 by mixed-effect ANOVA) (Fig. 3B). Despite these differences in pupation rates, the quantities of colony-forming bacteria in L4 larvae were not significantly different with 1:500 or 1:10^4^ EW dilutions or with LW at 7 or 10 dpe (Fig. 3C), suggesting that larvae develop similar bacterial loads despite different initial exposure doses. The lack of a difference in larval development rates at various dilutions of EW microbes, together with the lack of a difference in microbial levels across EW and LW despite augmented pupation in EW, supports specific microbes, rather than absolute microbial levels, water chemistry, or nutrient content, as a driver of the faster and more efficient development of mosquitoes reared in water from the environment than in water from the laboratory.

### Mosquitoes reared in environment water are less competent ZIKV vectors than mosquitoes reared in water from the laboratory.

**FIG 4:**
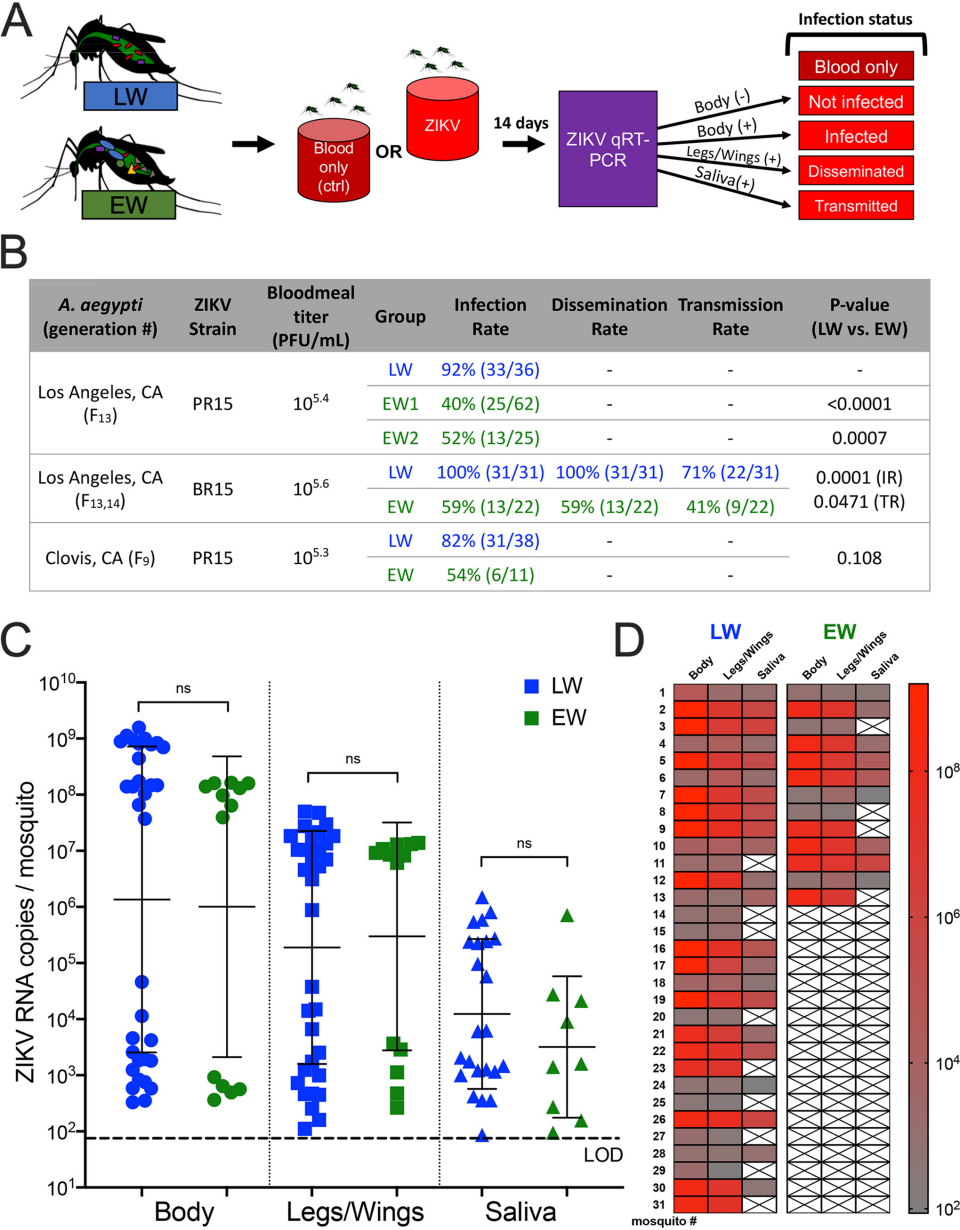
A. aegypti mosquitoes reared in environmental water are less competent ZIKV vectors than those reared in laboratory water. (A) Experimental overview of vector competence experiments showing control (no ZIKV) and ZIKV-exposed mosquitoes reared in either laboratory water (LW) or environmental water (EW) that were incubated for 14 days after bloodfeeding and then harvested to assess infection (bodies), dissemination (legs/wings), and transmission (saliva). (B) Summary table of infection, dissemination, and transmission rates. Transmission rate (TR) refers to the number of individuals transmitting from the total number of individuals that ingested a bloodmeal with ZIKV. Infection experiments were repeated once for replication. Transmission was assayed for the Los Angeles A. aegypti-ZIKV BR15 combination. *P* values were calculated with Fisher’s exact tests. IR, infection rate. (C) ZIKV RNA levels in Los Angeles A. aegypti mosquitoes infected with ZIKV BR15. Each symbol is for a single mosquito, and only mosquitoes that were ZIKV positive by qRT-PCR (*C_T_* < 40) are shown. Error bars denote the geometric means and standard deviations among positive individuals. The Mann-Whitney test was used. The dotted line denotes the average limit of detection, 65 ZIKV RNA copies/mosquito or saliva sample, across all qRT-PCR plates. (D) Heat map matching individual mosquitoes with their respective tissues, colored by ZIKV RNA levels. X in LW and EW saliva column to mosquito 13 indicates sample was not tested; only 13 mosquitoes were in EW, so X for mosquitoes 14 to 31 indicates samples do not exist.

We next assessed the influence of the source of rearing water on the vector competence of A. aegypti for ZIKV. LW- and EW-reared female adult mosquitoes were presented with matched ZIKV titers or blood only in artificial bloodmeals and then assayed 14 days after bloodfeeding using quantitative reverse transcription-PCR (qRT-PCR) to detect ZIKV RNA in bodies as a marker of infection, legs and wings to indicate dissemination, and saliva to assess transmission (Fig. 4A). No ZIKV RNA was detected in any mosquito that ingested blood only (data not shown). LW-reared mosquitoes were significantly more susceptible to infection and transmitted ZIKV at significantly higher rates than EW-reared mosquitoes (Fig. 4B). This pattern was observed with 2015 ZIKV strains from Puerto Rico and Brazil and two Californian A. aegypti lineages. Although infection, dissemination, and transmission rates were higher in LW-reared mosquitoes, the mean ZIKV genome copies in bodies, legs/wings, and saliva did not significantly differ between the LW- and EW-reared groups (Fig. 4C and Fig. S3A and B [showing additional experimental replicates that also revealed the same patterns]). Mosquitoes that contained >10^7^ ZIKV RNA copies in their body were more likely to contain detectable ZIKV RNA in saliva (likelihood ratios [LRs] of 3.48 in LW and 3.50 in EW) (Fig. 4D).

10.1128/msphere.00687-21.3 FIG S3 ZIKV RNA levels in individual mosquitoes that ingested bloodmeals containing 10^5.4^ PFU/ml (A) or 10^5.3^ PFU/ml (B) ZIKV or blood with no virus (mock) (summarized in Fig. 4B). (A) Colonized A. aegypti mosquitoes from Los Angeles, CA, were presented with PR15 ZIKV. (B) Colonized A. aegypti mosquitoes from Clovis, CA, were presented with ZIKV BR15. Mosquitoes with no detectable ZIKV RNA are reported at the limit of detection (LOD) of the assay, which averaged 10^1.3^ ZIKV genome copies/mosquito in both panels A and B. Download FIG S3, EPS file, 0.2 MB. Copyright © 2021 Louie and Coffey. 2021 Louie and Coffey. https://creativecommons.org/licenses/by/4.0/ This content is distributed under the terms of the Creative Commons Attribution 4.0 International license.

**FIG 5:**
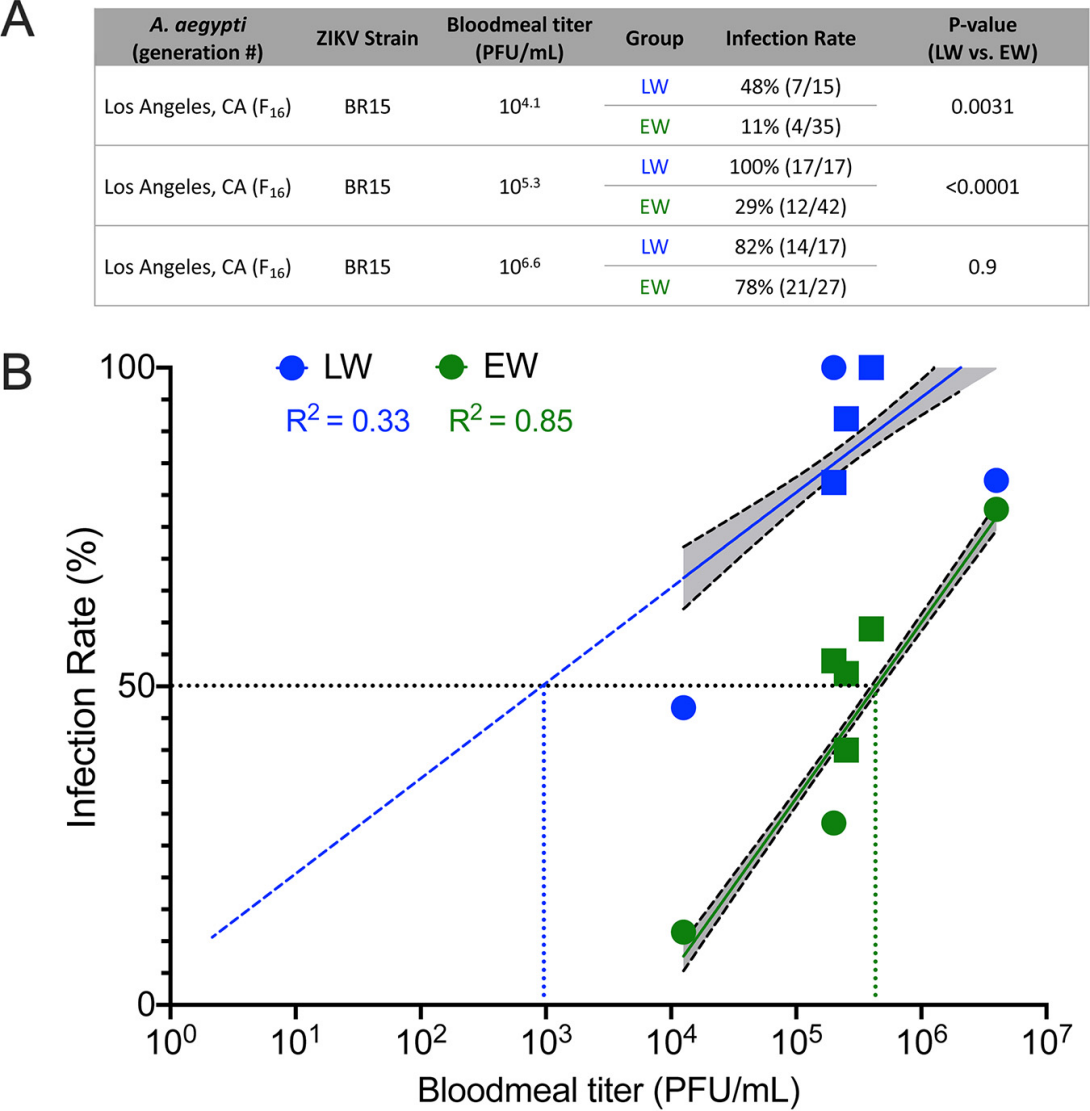
A. aegypti mosquitoes reared in environmental water require higher doses of ZIKV to become infected than those reared in laboratory water. (A) Summary of additional vector competence experiments using Los Angeles A. aegypti and ZIKV BR15 at three different bloodmeal titers. (B) Plot of infection rate versus bloodmeal titer. Squares represent the same data in Fig. 4B, and circles indicate the additional experiments in Fig. 5A. A best-fit nonlinear regression line with 95% confidence intervals (CIs) is shaded in gray. For LW, the slope is 14.9 (95% CI, 11.5 to 18.3), and the *y* intercept is 5.949 (95% CI, −12.57 to 24.44), and for EW, the slope is 27.6 (95% CI, 26.01 to 29.01), and the *y* intercept is −105.3 (95% CI, = −113.8 to −96.83).

To understand the dose response to ZIKV infection, A. aegypti mosquitoes reared in both water types were exposed to a range of bloodmeal titers below and above 10^5^ PFU/ml (Fig. 5A). LW-reared mosquitoes became infected at a significantly lower bloodmeal titer than EW-reared mosquitoes (*F* = 878 and *P* < 0.0001 for comparison of fits [slope and *y* intercept] by nonlinear regression) (Fig. 5B). The infectious bloodmeal titer that produced ZIKV infections in 50% of the cohort (50% infectious dose [ID_50_]) for LW-reared mosquitoes was 10^3.0^ PFU/ml, compared to 10^5.6^ PFU/ml for EW-reared mosquitoes, which represents a 400-fold difference. Mosquitoes reared in both water types followed a strong dose response to ZIKV infection (*R*^2^ = 0.33 for LW and 0.85 for EW by nonlinear regression). Together, these data demonstrate that laboratory water-reared mosquito colonies are more susceptible to ZIKV infection and transmission than mosquitoes reared in water from the environment. The higher ID_50_ of EW mosquitoes also suggests that these mosquitoes are less susceptible to infection by and transmission of ZIKV when ingesting a bloodmeal titer reflective of typical human viremia (53).

### Larval water source does not differentiate bacterial compositions between adult mosquitoes as much as bloodmeal status.

**FIG 6:**
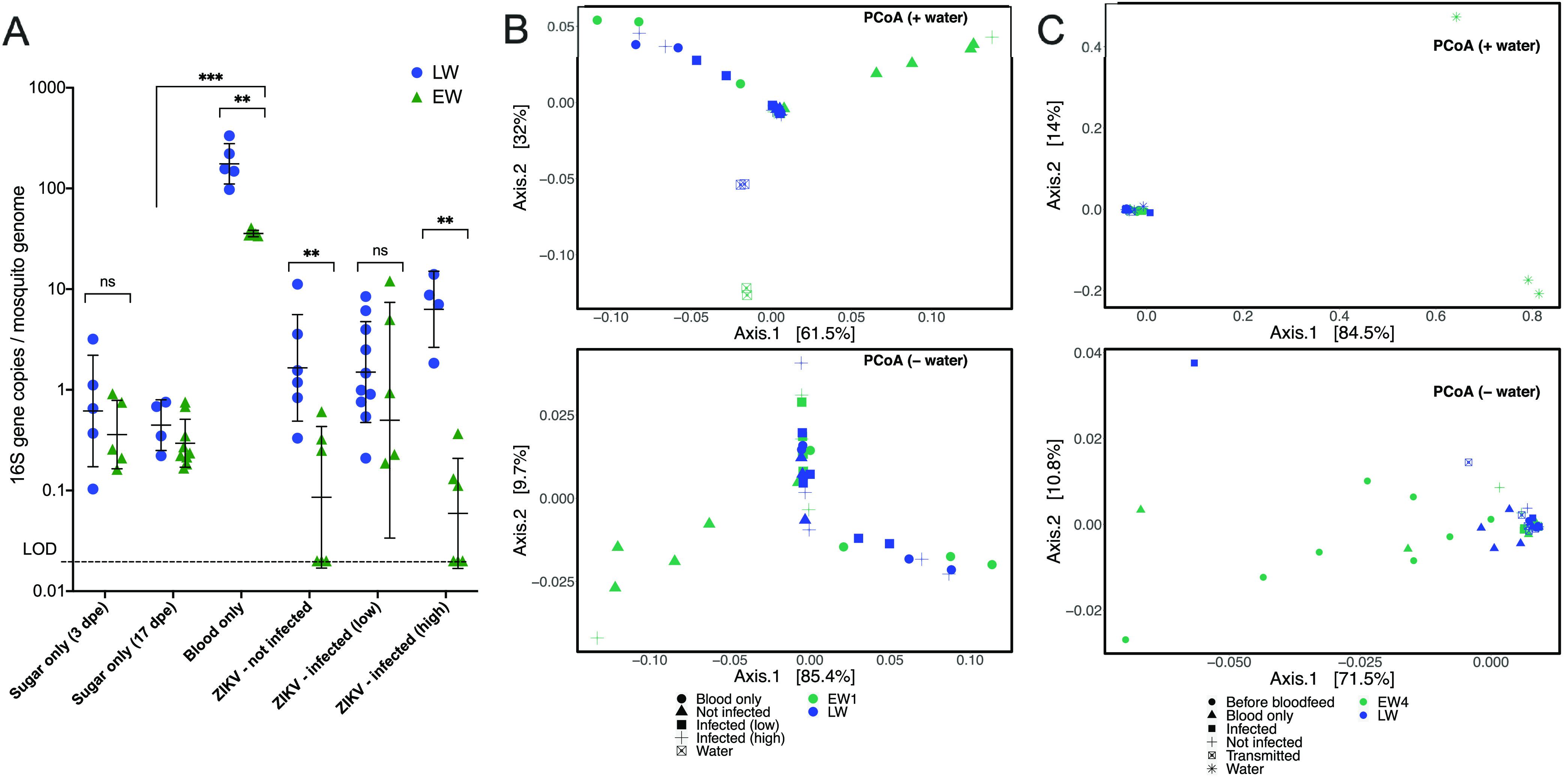
Microbial communities differ slightly by water type. (A) Bacteria in LW- or EW-reared A. aegypti mosquitoes exposed to sugar, blood only, or blood containing ZIKV quantified by 16S qPCR and normalized to the A. aegypti reference gene RPS17. Symbols refer to a single mosquito, and error bars denote the geometric means and geometric standard deviations. Statistically significant differences between LW and EW were determined by a Mann-Whitney test, while differences across treatments were determined by a Kruskal-Wallis test. Interaction effects between water type (LW and EW) and ZIKV infection states were investigated by two-way ANOVA with multiple comparisons, on log_10_-transformed values (*, *P* < 0.05; **, *P* < 0.01; ***, *P* < 0.001; ns, not significantly different at the level of a *P* value of 0.05). (B and C) Principal coordinates of UniFrac distances, colored by water type. (B) A cohort of Los Angeles A. aegypti mosquitoes presented with ZIKV PR15 (data set AM1019ZE) (Table 1); (C) another cohort of Los Angeles A. aegypti mosquitoes presented with ZIKV BR15 (data set AM820ZE) (Table 1). Top plots include mosquito and rearing water samples, while bottom plots have water samples omitted for higher resolution.

Although LW and EW A. aegypti mosquitoes that were not ZIKV exposed showed similar bacterial levels, we next questioned whether the same pattern would be observed in the context of ZIKV infection. Adult female mosquitoes reared in LW or EW that ingested ZIKV in bloodmeals were grouped into the following classes based on their infection outcomes: (i) not infected, where no ZIKV RNA was detected above the limit of detection of 65 ZIKV genomes/body; (ii) infected (low), defined as body titers of <10^6^ ZIKV genomes/body; or (iii) infected (high), defined as body titers of >10^6^ ZIKV genomes/body. The “high” and “low” infection states were defined based on the bimodal distribution of RNA levels observed in bodies (Fig. 4C). The reasoning for this grouping is that individuals with high ZIKV RNA levels in their bodies were more likely to have disseminated infections that lead to ZIKV RNA detection in saliva (Fig. 4D), a pattern also observed in previous studies with A. aegypti from the same source colonies and that used the same ZIKV strains as the ones in this study (23). Mosquitoes that fed on blood without ZIKV or that had been presented with only sugar water were included as controls. Prior to a bloodmeal, where mosquitoes had been exposed to only sugar at 3 dpe, both LW and EW females had bacterial quantities in their bodies that were not significantly different (*P* = 0.5476 by a Mann-Whitney test), and the bacterial load was low (Fig. 6A). Ingestion of blood resulted in a 50- to 100-fold increase in bacterial levels in both groups compared to unfed mosquitoes of the same age (*P* = 0.0005 by a Kruskal-Wallis test [adjusted *P* = 0.012 for LW and adjusted *P* = 0.0212 for EW by multiple comparisons]). Bloodfed LW mosquitoes contained significantly higher bacterial levels than EW mosquitoes (*P* = 0.0079 by a Mann-Whitney test). Regardless of the infection outcome, both LW and EW mosquitoes that ingested ZIKV showed lower bacterial levels than the blood-only groups (*F* = 33.41 and *P* < 0.0001 for infection state and *F* = 40.30 and *P* < 0.0001 for water type, by two-way ANOVA). Moreover, two-way ANOVA on bloodfed mosquitoes detected a significant interaction between water type and ZIKV infection state (interaction *F* = 4.6 and *P* = 0.0032). LW mosquitoes that were not ZIKV infected or that were infected at high levels contained higher bacterial levels than EW mosquitoes (*P* = 0.0043 and *P* = 0.0095, respectively, by a Mann-Whitney test).

Next, we examined the bacterial compositions of LW and EW mosquitoes, reasoning that the type of bacteria may influence vector competence more than the total bacterial load. We compared the relative bacterial abundances and taxonomic diversities of LW and EW adult mosquitoes as well as across mosquitoes that exhibited differential ZIKV infection states from two replicate vector competence experiments. Totals of 1,077 and 221 ASVs were identified in the first and second vector competence experiments, respectively (AM1019ZE and AM820ZE) (Table 1), most of which were detected in the rearing water (Fig. S4). EW1 and EW4 denote environmental water samples collected from the same cemetery in different months that were used for separate rearing experiments to account for the temporal variation in microbes within the same environment. Comparing EW1 and LW, we detected no significant differences in the clustering of samples by water type; however, when we compared EW4 and LW, we detected a difference in clustering (*F* = 2.52 and *P* = 0.043 [Adonis] and *F* = 6.91 and *P* = 0.018 [Betadisper] by permutational multivariate analysis of variance [PERMANOVA]). The disparity in clustering patterns across different EW sample collections may be due to differences in community distributions in EW4 versus LW arising from the low number of ASVs, where most EW4 and LW mosquitoes in the AM820ZE data set were dominated by Pseudomonas (Fig. 6C, Fig. S4C, and Fig. S6C). Despite the identification of different ASVs between experimental iterations, EW had higher bacterial diversity and evenness than LW (*F* = 17.25 and *P* < 0.0001 [richness] and *F* = 12.8 and *P* < 0.0001 [Shannon] by one-way ANOVA) (Fig. S4). When we compared mosquitoes only (and not their rearing water), the bacterial compositions between LW and EW mosquitoes were slightly different, as shown by partial sample overlaps, although clustering was not significantly different (*F* = 1.87 and *P* = 0.111 [Adonis] and *F* = 0.15 and *P* = 0.707 [Betadisper] by PERMANOVA) (Fig. 6B, bottom). Several EW mosquitoes that were refractory to ZIKV infection clustered together; these individuals had increased proportions of *Asaia* and *Flavobacterium* and significantly reduced proportions of *Rhodovarius*, *Micrococcus*, and *Neochlamydia* bacteria compared to infection-competent individuals, as determined by DESeq2 analysis and random forest modeling (Fig. S5A and Fig. S6A and B). The overall contribution of water type to differences in bacterial compositions across individual mosquitoes of either bloodfed status was 22%. Whether mosquitoes ingested ZIKV and whether mosquitoes that ingested ZIKV became infected were also important variables that explained 27% and 34%, respectively, of the differences in bacterial compositions across groups (Table 2). EW mosquitoes that ingested blood with ZIKV had a reduced abundance of *Serratia* compared to EW mosquitoes that ingested blood only (Fig. S5B and Fig. S6B). For both data sets, there were no significant differences in bacterial compositions in LW mosquitoes that ingested blood only or ZIKV; this may be an artifact of LW mosquitoes possessing few bacterial taxa such that differential abundances could not be detected (Table 2 and Fig. S6A and B). Additionally, no differences in bacterial composition were detected between mosquitoes with high and low levels of ZIKV RNA or between mosquitoes that were infected and those that transmitted (Fig. 6C and Table 2). Taken together, EW-reared adult females harbor different microbiota when reared in the same water source collected at different times. This suggests that despite differences in microbiota, adult female A. aegypti mosquitoes exhibit consistently reduced vector competence for ZIKV when reared in environmental water compared to laboratory water.

**TABLE 2:** Summary of contributions to microbial compositional differences by mosquito variable^*a*^

Group	variable compared 1	variable compared 2	variable contribution	p-value
-	EW1	LW	22%	0.008**
EW1	ZIKV-exposed	Blood only	27%	0.019*
	Infected	Not infected	34%	0.011*
	Infected(low)	Infected(high)	10%	0.676
LW	ZIKV-exposed	Blood only	13%	0.619
	Infected	Not infected	8%	0.438
	Infected(low)	Infected(high)	10%	0.676
-	EW4	LW	5%	0.012*
EW4	Before bloodfeed	After bloodfeed	11%	0.074
	ZIKV-exposed	Blood only	12%	0.01**
	Infected	Not infected	8%	0.463
	Infected	Transmitted	9%	0.963
LW	Before bloodfeed	After bloodfeed	6%	0.692
	ZIKV-exposed	Blood only	9%	0.037*
	Infected	Not infected	7%	0.319
	Infected	Transmitted	5%	0.693

a Variable contribution, the percentage of the variance between samples associated with the metadata, was calculated using constrained analysis of principal coordinates. Statistical tests were performed by permutational ANOVA (*, *P* < 0.05; **, *P* < 0.01).

10.1128/msphere.00687-21.4 FIG S4 Alpha diversity of EW1, EW4, and LW mosquitoes. Download FIG S4, EPS file, 0.7 MB. Copyright © 2021 Louie and Coffey. 2021 Louie and Coffey. https://creativecommons.org/licenses/by/4.0/ This content is distributed under the terms of the Creative Commons Attribution 4.0 International license.

10.1128/msphere.00687-21.5 FIG S5 Differentially abundant taxa determined by DESeq2. Each dot represents an ASV from the respective genus (*y* axis) that was differentially abundant among mosquitoes according to infection status or water type, which are separated by the vertical red line. ASVs to the left of the dividing line were more abundant in the left group, while ASVs to the right of the dividing line were more abundant in the right group. Bacterial taxa from DESeq2 were significantly different if the adjusted *P* value cutoff (alpha) was below 0.05. No differentially abundant ASVs were found between not-infected/infected and blood-only/ZIKV-exposed mosquitoes in LW. Download FIG S5, EPS file, 0.2 MB. Copyright © 2021 Louie and Coffey. 2021 Louie and Coffey. https://creativecommons.org/licenses/by/4.0/ This content is distributed under the terms of the Creative Commons Attribution 4.0 International license.

10.1128/msphere.00687-21.6 FIG S6 Random forest modeling of significant bacterial taxa. The top 5 to 7 predictors of mosquito groups were plotted (A), and relative abundances of the top 5 to 7 selected predictors were plotted using ggplot2 (B). “Ctrl” indicates mosquitoes that ingested blood only but no ZIKV. Download FIG S6, EPS file, 0.5 MB. Copyright © 2021 Louie and Coffey. 2021 Louie and Coffey. https://creativecommons.org/licenses/by/4.0/ This content is distributed under the terms of the Creative Commons Attribution 4.0 International license.

## DISCUSSION

Here, we show that microbial diversity stemming from different water sources used to rear larvae in a laboratory environment modifies the vector competence of A. aegypti for ZIKV. Reduced vector competence in environmental water-reared A. aegypti was consistently observed using two lineages of Californian A. aegypti and two epidemiologically relevant ZIKV strains. These results suggest that modification of A. aegypti developmental conditions to reflect environmental water compared to laboratory tap water, which is conventional, decreases laboratory infection and, potentially, transmission rates for ZIKV. The use of laboratory water to rear larvae likely leads to overestimates of the transmission potential of ZIKV vectors in the environment. This pattern may apply to other vector-virus pairings as well, and future research should address this question. Due to the wide range of urban environments in which A. aegypti larvae develop, watering cans, bromeliads, potted plants, and abandoned tires, etc., and because each environment contains its own microclimate with unique microbial composition and nutrient content, studying whether larval development in different water containers also reduces vector competence would be of great interest.

Differences in pupation kinetics between EW- and LW-reared mosquitoes indicate that the type of bacteria, but not bacterial abundance, impacts the success of mosquito larval development; this mostly agrees with previous studies on gnotobiotically reared larvae with bacteria and yeast of similar densities (54). Since pupation in insectary environments typically occurs before 8 days, the earliest time that we observed pupation for LW larvae, we cannot exclude the presence of growth-inhibiting microbes in LW that were absent from EW larvae that pupated at higher rates and with faster kinetics. Alternately, by sterilizing the fish food to ensure that the microbes were derived from the water only, we may have hindered pupation rates and kinetics, where nonsterilized food, as is conventionally used, may be a requisite for rapid larval development. Our observation of wide variability in microbial contents in experiments using different collections of environmental water, but which all yielded 100% pupation success, suggests that there is likely functional redundancy in microbes needed to nutritionally support larval growth and stimulate pupation. While the bacterial diversities of laboratory and environmental water from natural mosquito larval habitats were different, bacterial taxonomic differences within mosquitoes reared in water from these respective sources were more subtle. This suggests that mosquitoes may harbor a relatively low number of species in a “core” microbiome (55), possibly explaining the low number of bacterial species detected and the lack of shared species across experimental replicates. In concordance with previous A. aegypti microbiome studies, we observed high relative abundances of *Proteobacteria* and *Bacteroidetes* in adult mosquitoes (38, 42). At the genus level, most adult mosquitoes were dominated by *Asaia*, *Flavobacterium*, *Elizabethkingia*, and Pseudomonas bacteria. These bacteria were also found in small quantities in their rearing water, suggesting that they are likely environmental in origin, except for *Elizabethkingia*, which was also detected in surface-sterilized eggs. Because the same ASVs matching *Elizabethkingia* were also identified in surface-sterilized eggs, the origin of *Elizabethkingia* in mosquitoes in this study cannot be determined. Bacteria from this genus are present in the environment, larvae, newly emerged adults, and also reproductive tissues of *Aedes* species mosquitoes (56) but have not yet been reported in eggs.

By varying the source of larval rearing water, we aimed to modify the microbiota of A. aegypti with the premise that mosquito microbes are acquired through the environment and especially larval water. We therefore expected that a sterile sugar diet and a single artificial bloodmeal provided to adults would narrow the microbial input of the mosquitoes to reflect larva-acquired microbes from the rearing water. While we detected differences in the microbiota in LW- versus EW-reared mosquitoes, the microbiota was more different between control bloodfed and ZIKV-bloodfed groups. Other studies have also measured strong relationships between bloodmeal status and microbiome composition (57, 58), with some showing greater differences in the expression of A. aegypti genes in mosquitoes that bloodfed than between axenic and conventionally reared mosquitoes (59). A functional limitation of this and previous work is the inability to account for all microbial sources in adult mosquitoes stemming from their natural field environment, including microbes acquired during sugar feeding of adults on flora.

Despite the lack of reproducible changes in the species composition of bacteria in adult A. aegypti mosquitoes reared in different aquatic environments, we observed a substantial effect on vector competence, where EW-reared mosquitoes exhibited lower infection and transmission rates than LW-reared mosquitoes. As this is the first study examining the microbiota of Californian A. aegypti and one of the few mosquito studies using ASVs instead of operational taxonomic units (OTUs), where ASVs are gaining favor over OTUs due to their increased taxonomic resolution as well as their consistent labeling (60), direct comparisons to other A. aegypti microbiome studies should be made with caution. In addition to a “core microbiome” effect on mosquito vector competence, there could also be functional redundancy in the effects of the microbiota on mosquito physiology. Despite microbial variability in rearing water and mosquitoes observed in our experimental replicates, the increased infection and transmission of ZIKV by LW- compared to EW-reared mosquitoes was reproducible. Although we studied only fully bloodfed mosquitoes for ZIKV vector competence assays, we cannot exclude the possibility that EW mosquitoes ingested lower bloodmeal volumes than LW mosquitoes, which may have resulted in lower infection rates. However, even a 2-fold difference in the ingested viral dose is not expected to substantially impact infection rates since mosquito dose-response studies typically follow a log-linear dose-response relationship, which surpasses the likely magnitude of the variance in the bloodmeal volume. Finally, while the transmission rates by EW mosquitoes were demonstrated to be lower than those of LW mosquitoes in Los Angeles A. aegypti mosquitoes with ZIKV strain BR15 (Brazil 2015), it is not certain whether the reduced transmission potential in EW mosquitoes is true for ZIKV in A. aegypti in general. Since transmission was assayed in only one A. aegypti-ZIKV pairing, replication of this result in other A. aegypti colony-ZIKV strain combinations would be needed to definitively confirm the reduced transmission of multiple ZIKV strains by A. aegypti.

The overall reduction of bacterial levels in ZIKV-exposed mosquitoes relative to nonexposed mosquitoes suggests that ZIKV infection negatively impacts the mosquito microbiota. This could be due to interactions between the mosquito antiviral immune response and a generalized antimicrobial effect that indirectly kills bacteria within the mosquito gut. Another study with Brazilian A. aegypti found enrichment of *Rhodobacteraceae* and *Desulfuromonadaceae* in response to ZIKV infection (61), the former of which were not differentially abundant in our data set, while the latter were absent from both our mosquitoes and rearing water. These discrepancies imply that bacterium-mosquito interactions during ZIKV infection are region specific. Previous work on A. aegypti innate immunity implicated a link between antiviral and antibacterial immune responses to infection (48, 62, 63). For example, the Toll pathway, which recognizes bacterial cell walls in insects, also modulates responses to DENV infection (47). This implicates a nonspecific pan-arboviruses effect where elevated immune responses to the resident microbiota confer resistance to infection. Furthermore, additional life-history traits like adult body size are influenced by larval water conditions (16, 50), implicating a physiological modification that may indirectly result from microbial exposures of larvae. Moreover, gut microbes play a nutritional role in mosquito symbiosis (44, 59), and larval nutrition impacts mosquito size and development (64), although the role of size in the vector competence of A. aegypti and DENV and *Culex* species mosquitoes and West Nile virus is controversial (65–67). Carryover effects (49) of larval exposure to isolates of *Flavobacterium*, *Lysobacter*, *Paenibacillus*, and *Enterobacteriaceae* on adult lipid metabolism and DENV infection in A. aegypti corroborate our observations that bacterial exposure during the larval stage can influence adult mosquito traits. Interestingly, oral treatment of adults with antibiotics did not change their vector competence for ZIKV, suggesting that these carryover effects from larvae could become fixed after maturity (68). The influence of bacteria known to impact vector competence in a monoculture in the context of the complex microbial community should be a target of future research.

## MATERIALS AND METHODS

### Biosafety.

All ZIKV experiments were conducted in a biosafety level 3 laboratory and were approved by the University of California, Davis, under biological use authorization number R1863.

### Mosquitoes.

Two sources of A. aegypti mosquitoes were used in this study. A. aegypti mosquitoes were field collected as larvae in Los Angeles, CA, or as eggs in Clovis, CA, in 2016 and reared under standard insectary conditions for several generations until F_13–16_ and F_9_ eggs, respectively, were collected for use. Adults were morphologically identified by personnel trained in recognizing A. aegypti. Insectary conditions during the laboratory colonization process were 26°C, 80% humidity, and a 12-h/12-h light/dark cycle, with larvae maintained in 1 liter of deionized water (diH_2_O) at 200 to 400 larvae per pan and provided 1 pinch of fish food (Tetra, Melle, Germany) every other day until pupation. Adults were maintained in 30- by 30- by 30-cm mesh cages (BugDorm; Megaview Science, Taiwan) with constant access to 10% sucrose, all under septic conditions.

### Mosquito rearing.

Urban-adapted A. aegypti larvae are known to develop within open containers, including cemetery headstones, plant pots, rain barrels, abandoned tires, and bromeliads, which tend to accumulate nutrients and organic matter (17, 69–71). Outdoor and laboratory water sources were used in this study (see Table S1 in the supplemental material). For the laboratory water, ethanol-cleaned plastic trays were filled with 1 liter of laboratory tap diH_2_O in an insectary. Environmental water consisted of 2 to 3 liters per collection of stagnant water from headstone receptacles in Davis Cemetery (Davis, CA) after rainfall. Separate water collections were conducted prior to each experiment to encompass variation in outdoor environmental conditions over time. Collected water was used for two purposes, (i) as rearing water and (ii) pelleted to isolate microbes prior to inoculation in laboratory tap water, in separate experiments. The cemetery water was filtered through 1-mm mesh to remove insects, larvae, and large particulates and then centrifuged at 3,000 × *g* for 30 min to pellet microbes. The supernatant was discarded, and pellets were washed with sterile 1× phosphate-buffered saline (PBS; Thermo Fisher Scientific, Emeryville, CA) three times prior to creating glycerol stocks of pelleted microbes that were frozen for later use. Pellet aliquots were also plated onto LB agar plates in parallel (Sigma-Aldrich, St. Louis, MO) to estimate live bacterial quantities prior to freezing at −80°C.

10.1128/msphere.00687-21.7 TABLE S1 Water sources, collection dates, and data references for A. aegypti mosquitoes used in rearing experiments. LW, laboratory water; EW, environmental water. Download Table S1, EPS file, 0.6 MB. Copyright © 2021 Louie and Coffey. 2021 Louie and Coffey. https://creativecommons.org/licenses/by/4.0/ This content is distributed under the terms of the Creative Commons Attribution 4.0 International license.

Mosquito eggs were surface sterilized by submerging in 5% bleach (Clorox, Oakland, CA) for 10 min, washed twice in 70% ethanol (Thermo Fisher Scientific, Emeryville, CA), and dried for 10 min before hatching in diH_2_O. A PBS wash on a subset of eggs after surface sterilization was cultured on LB medium to confirm the removal of live bacteria from egg surfaces. Hatching was stimulated either by a pinch of active dry yeast (Red Star Yeast, Milwaukee, WI) in larval water or by inducing negative pressure (Rocker 400 vacuum pump; Sterlitech Corp., Kent, WA) to reduce the dissolved oxygen content for 30 min. A total of ∼2,500 larvae were transferred to six 1-liter pans to achieve a density of 400 to 500 larvae/pan. Food was prepared in agarose plugs that were made by mixing 1% agarose (Sigma-Aldrich, St. Louis, MO) with pulverized fish food (final concentration of 100 g/liter, or 10% [Tetra, Melle, Germany]) and rodent chow (final concentration of 80 g/liter, or 8% [Teklad Global 18% protein rodent diet; Envigo, Indianapolis, IN]), which was then autoclave sterilized before casting into 12-well plates, a modification of a previously described approach (44) for standardizing the larval diet. One plug was fed to larvae in each pan every other day. Pupae were counted once daily and transferred into plastic dishes containing sterile diH_2_O within 30-cm^2^ cloth cages. Once cages reached a mosquito density of about 500, adult females were transferred in batches of 100 to 32-oz plastic containers (Amazon, Seattle, WA) for vector competence experiments. Larval development experiments were repeated twice. Larval trays and adult mosquitoes were maintained at 26°C with 80% humidity and a 12-h/12-h light/dark cycle for the duration of the experiment. All trays and adult mosquitoes were housed in the same incubator. Adult mosquitoes were provided constant access to filter-sterilized 10% sucrose (Thermo Fisher Scientific, Emeryville, CA).

### Virus sources and titrations.

Two Asian-lineage ZIKV strains were used: PR15 (Puerto Rico 2015) (PRVABC59 [GenBank accession number KX601168]) and BR15 (Brazil 2015) (SPH2015 [GenBank accession number KU321639]), both of which were isolated from human serum and passaged 3 times in Vero cells (ATCC CCL-81; ATCC, Manassas, VA) before freezing in stocks. Stocks were titrated on Vero cells prior to bloodmeal presentation to confirm titers. The remaining bloodmeals were recovered after presentation to mosquitoes, frozen at −80°C, and back-titrated by a plaque assay on Vero cells to confirm the administered dose. For titrations, bloodmeals were serially diluted 10-fold in Dulbecco’s modified Eagle’s medium (DMEM), inoculated into one well, and incubated for 1 h at 37°C in 5% CO_2_ with rocking every 15 min to prevent cell death due to desiccation. After 1 h, 3 ml of 0.5% agarose (Thermo Fisher Scientific, Emeryville, CA) mixed with DMEM supplemented with 2% fetal bovine serum (FBS) and penicillin-streptomycin (Thermo Fisher Scientific, Emeryville, CA) was added to each well to generate a solid agar plug. The cells were incubated for 7 days at 37°C in 5% CO_2_, after which they were fixed with 4% formalin (Thermo Fisher Scientific, Emeryville, CA) for 30 min, plugs were removed, and wells were stained with 0.025% crystal violet (Thermo Fisher Scientific, Emeryville, CA) in 20% ethanol to visualize and quantify plaques. ZIKV bloodmeal titers were recorded as the reciprocal of the highest dilution where plaques were noted and are represented as PFU per milliliter of blood.

### Zika virus vector competence experiments.

Stock ZIKV inocula in DMEM, or DMEM with no virus as a control, were mixed at a 1:10 or 1:20 ratio with fresh heparinized sheep blood (HemoStat Laboratories, Dixon, CA) to achieve ZIKV titers of 10^4^ to 10^6^ PFU/ml for each experiment. Bloodmeals were presented to 200 to 300 female A. aegypti mosquitoes at 3 to 5 days posteclosion in cohorts of 100 per container with 2to 3 containers per group, 24 h after sugar withdrawal. Bloodmeals were presented for 60 min through a collagen membrane that was rubbed with an artificial human scent (BG-Sweetscent mosquito attractant; Biogents USA) and heated to 37°C in a membrane feeder (Hemotek Ltd., Blackburn, United Kingdom). Fully engorged females (40 to 70 per group for each experiment) with blood in their abdomens visible at a ×10 magnification were cold anesthetized by holding for 4 min at −20°C, sorted into clean plastic containers at a density of 20 to 30 mosquitoes per container, and held at 28°C with 80% humidity and a 12-h/12-h light/dark cycle for 14 days, with constant access to filter-sterilized 10% sucrose. Fourteen days after bloodfeeding, mosquitoes were cold anesthetized and held immobile on ice. Legs and wings were removed before collection of the expectorate for 20 min into capillary tubes containing PBS (23). Each capillary tube was placed into a 1.5-ml tube containing 250 μl PBS and centrifuged at 8,000 × *g* for 1 min to recover saliva. Legs/wings and bodies were placed into 2-ml tubes (Thermo Fisher Scientific, Emeryville, CA) containing 500 μl PBS and a 5-mm glass bead (Thermo Fisher Scientific, Emeryville, CA). Surgical tools were washed once in Cavicide and twice in 70% ethanol between each dissection to minimize cross-contamination. For samples where microbial DNA from mosquito bodies was also analyzed in addition to viral RNA, the bodies were also washed twice in 70% ethanol and once in PBS prior to dissection to remove microbes on the surface of mosquitoes. Tissues were homogenized at 30 Hz for 10 min in a TissueLyzer (Retsch, Haan, Germany) before extracting viral RNA using a MagMax viral RNA extraction kit (Thermo Fisher Scientific, Emeryville, CA), into 60 μl elution buffer according to the manufacturer’s recommendations. Detection and quantification of viral RNA in mosquito tissues and saliva were performed by quantitative reverse transcription-PCR (qRT-PCR) using TaqMan Fast virus 1-step master mix and a ZIKV-specific primer set (ZIKV 1086F/1162c) (probe, ZIKV 1107-FAM [6-carboxyfluorescein]) using established methodologies (23, 72). Cycle threshold (*C_T_*) values from qRT-PCR were converted to RNA genome copies using standard curves established with known ZIKV RNA concentrations. Samples were assayed in technical duplicates and averaged together after conversion to RNA copies per milliliter. The limit of detection (LOD) was calculated from the standard curve linear regression line where the *C_T_* value was 40; samples that did not yield a detectable *C_T_* of <40 were reported at the LOD. Infection experiments were each repeated once.

### 16S amplicon sequencing and bioinformatics.

DNA from individual mosquitoes (5 to 8 biological replicates per treatment) was extracted with a Quick-DNA Tissue/Insect Microprep kit (Zymo Research, Irvine, CA, USA), according to the manufacturer’s instructions, and eluted in 30 μl elution buffer. DNA from larval water and bloodmeals was extracted with a DNeasy blood and tissue kit (Qiagen, Hilden, Germany) according to the manufacturer’s recommendations. DNA extracted from individual mosquitoes was PCR amplified in either the V3-V4 (73) or solely the V4 (74) hypervariable region of the 16S rRNA gene. The presence and size of amplicons were confirmed by gel electrophoresis using a DNA ladder to identify the amplicon size (GeneRuler 1-kb Plus; Thermo Fisher Scientific, Emeryville, CA). Negative controls, including DNA extraction controls (extraction protocol with sterile PBS) and PCR controls (PCR with molecular-grade H_2_O), were included in each library preparation. 16S amplicon libraries were prepared by the addition of Nextera XT index kit v2 set A adapter sequences (Illumina, San Diego, CA), which were cleaned using Kapa Pure beads (Roche, Basel, Switzerland), quantified by a Qubit double-stranded DNA (dsDNA) high-sensitivity (HS) assay (Thermo Fisher Scientific, Emeryville, CA), pooled to equimolar concentrations of 5 nM per sample, and sequenced at the University of California, Davis, DNA Core Laboratory using the Illumina MiSeq PE250 platform.

The bacterial composition of individual mosquitoes from different water types and that exhibited different ZIKV infection statuses was assessed by bioinformatic analysis of the 16S rRNA amplicon. Paired-end reads were filtered, trimmed, and processed using the DADA2 pipeline (package version 1.16.0) according to the recommended workflow (75, 76), which was handed to phyloseq (version 1.32.0) (77). Sequences were grouped into amplicon sequence variants (ASVs), a proxy for species (60), and assigned taxonomy using the Silva v132 reference database (78). Assigned taxa were filtered to remove environmental contaminants and sequencing artifacts. Contaminant and artifact ASVs were identified and removed if sequences were also present in the negative controls (DNA-extracted nuclease-free H_2_O) or if reads aligned with “arthropod,” mitochondrial, or chloroplast sequences.

Microbial ecology analyses were conducted using the R packages phyloseq (version 1.32.0) and vegan (version 2.5.7) (77, 79). To determine whether ASVs showed differential abundances across samples, differential expression analysis was conducted using DESeq2 (80). Random forest modeling was used to predict ASVs that distinguish mosquito cohorts, using the randomForest package (81). Sample reads were scaled to an even depth (mean number of reads per sample) prior to all analyses.

### Microbial quantification.

Both culture-dependent and culture-independent assays were conducted in parallel to quantify live and total bacterial loads in mosquitoes and their rearing water. Culture-dependent quantification of microbes was performed by culturing 40 μl of rearing water or 40 μl of 10-fold serial dilutions from individual mosquitoes (3 to 5 per treatment) homogenized in 500 μl PBS on LB plates at 37°C for 5 days. Plated dilutions that yielded distinct, countable colonies were enumerated for each mosquito sample. Each sample was plated in technical triplicates, and the mean colony count is reported. Culture-independent quantification of bacteria was performed by SYBR green real-time PCR (Thermo Fisher Scientific, Emeryville, CA) to amplify the 16S rRNA gene in samples from mosquitoes and water (5 to 10 per treatment). Bacterial culturing and quantitative PCR (qPCR) of mosquitoes were repeated twice for each rearing experiment. The mosquito data were normalized to an A. aegypti reference ribosomal protein S17 (RPS17) gene (82).

### Statistical analyses.

Differences in pupation kinetics were determined by mixed-effect ANOVAs with repeated measures. Bacterial abundance differences between groups were determined by either Mann-Whitney or Kruskal-Wallis tests. For 16S amplicon sequencing, differences in microbial communities were assessed using principal-coordinate analysis (PCoA) of weighted UniFrac distances and tested for significance by permutational multivariate analysis of variance (PERMANOVA). Quantification of the contribution of each variable to differences in microbial communities was conducted using constrained analyses of principal coordinates with the same UniFrac distances as those in the PCoA analyses.

Vector competence was assessed by quantifying infection, dissemination, and transmission rates, calculated as the number of individual bodies, legs/wings, or expectorates, respectively, that yielded detectable ZIKV RNA divided by the total number of individuals that ingested blood. The magnitude of ZIKV RNA in individual mosquito tissues is also reported. Differences in infection, dissemination, and transmission rates between mosquito groups were determined using Fisher’s exact tests, and differences in RNA levels were assessed by Mann-Whitney tests. Calculation of the 50% infectious dose (ID_50_) was performed using the nonlinear regression dose curve for LW and EW groups. All statistical analyses were performed using GraphPad Prism 9.0.2 (GraphPad Software, San Diego, CA).

### Accession number(s).

Raw sequencing data are available from the NCBI Sequence Read Archive under BioProject accession number PRJNA750810.
